# Remarks upon an "Amended Bill" for Regulating Mad-Houses

**Published:** 1814-07

**Authors:** C. Harrison


THE
### Medical and Physical Journal.

1 OF VOL. XXXII.] JULY, 1814. [NO. 185.

"For many fortunate discoveries in medicine, and for the detection of nume-
"rous errors, the world is indebted to the rapid circulation of Monthly
"Journals; and there never existed any work to which the faculty in
"EUROPE and AMERICA were under deeper obligations than to the
"Medical and Physical Journal of London, now forming a long, but an
"invaluable series."—RUSH.

*For the Medical and Physical Journal.*

### *Remarks upon an "Amended Bill" for regulating Mad-houses; by* DR. HARRISON.

I HAD an opportunity this evening to peruse an "amended bill" lately introduced into the House of Commons, and which will doubtless soon become an act of the legislature, unless its progress be arrested by the active and spirited co-operation of the medical faculty in all parts of the island. It is denominated a "Bill to repeal an Act made in the 14th year of his present Majesty, for regulating Mad-houses, and for making other Provisions and Regulations in lieu thereof."

To me it appears to be more a bill to extend the power, and augment the revenues, of the Royal College of Physicians, London, than to promote the comfort or contribute to the recovery of insane persons. It is on this ground that I wish to call the attention of your numerous readers to the subject and provisions of the intended enactment. Under it the College are alone to possess the right of granting licenses to keepers of lunatic institutions "in the cities of London and Westminster, and within seven miles of the same, and within the county of Middlesex." They are moreover to be the only visitors of lunatic asylums in the above districts. Here then is a source of great power to the College of Physicians in the vicinity of the capital, and which is deserving of attentive consideration from the metropolitan faculty. In other parts of England the licenses are to be granted by justices at quarter sessions, but an account of them must be forwarded to the Royal College; and the keepers of lunatic asylums are directed under heavy penalties speedily to transmit to the College the names of all insane persons committed to their care.

It will be seen, on perusal of the bill, that the College will derive great influence from it in various ways, and in every part of England. This increase of authority I view with alarm, constituted as the College now is, and could wish to hear that preparations are making to exhibit the College as it is really constituted, at the bar of the House of Commons, that the public may be no longer amused with sounding titles. It will be seen, 1st, on examining the bill, that the College as a body, and some of its members, will individually derive considerable emolument under the act. This they will do by the issue of annual licenses to keep houses for lunatics. These vary from 10*l.* to more than 60*l.*, each arising according to the number of inmates. They are moreover to receive two weeks pay from every lunatic patient, and also two weeks salary from all keepers of lunatic houses; and in every succeeding year a moiety of the above sums. They are likewise to receive a moiety of all penalties incurred under this act in London, Westminster, and the county of Middlesex. Now when we consider the amount of licenses issued, the number of lunatics lodged in asylums, and the quantity of keepers employed, the aggregate revenue to the College obtained in this way will amount to a very large sum indeed.

2dly, Nor have they been unmindful of power in the new bill. The jurisdiction of the College is to be extended under it to a greater distance round the cities of London and Westminster than formerly, besides which it is to comprehend the whole county of Middlesex. With a view to increased power, the visiting physicians of all lunatic abodes must be graduates of the English universities, or licensed to practise physic by the College of Physicians. Still further to promote the same object, "no keeper of any house, &c. shall admit any lunatic without a written certificate of some physician, being a graduate of Oxford or Cambridge, or licensed by the College of Physicians in London, or a member of the College of Surgeons in London, or of the Society of Apothecaries in London actually practising as an apothecary." In designating the medical men who are to visit lunatic institutions, and to grant licenses of admission to patients, the College has insidiously contrived to serve its own body and the other incorporations, to the exclusion of every other practitioner. In the former act, all physicians, surgeons, and apothecaries could grant licenses of admission, and certainly no good reason can now be given for excluding them, them, in favour of doctors and bachelors of medicine, whose education has been conducted in universities notoriously destitute of medical instruction. Hitherto an apprenticeship to pharmacy has been deemed a sufficient education for apothecaries, though certainly it is quite inadequate to form the modern apothecary, or general practitioner. Nor must the uninformed be deceived by the examinations at Apothecaries' Hall, which are always purely pharmaceutical, and therefore should not entitle its members to any preference over their unadmitted brethren as medical practitioners. The exception appears partial and illiberal. In London the faculty may have private inducements to enter the corporate bodies; in the provinces no additional advantages being derived from admission, they have been so much neglected, that many counties do not contain a single fellow or licentiate of the College of Physicians, or a member of the Company of Apothecaries, to carry the provisions of the intended act into effect. In such cases, what can be done? Is a visiting physician to be sent down from the Royal College, and are the lunatics to be transported into other counties, or even to London, to obtain the required certificates?

It is obvious that these restrictive clauses cannot be carried into effect without incurring in many instances an enormous expense, and great public inconvenience. Some of the brightest luminaries in medicine will be disqualified, and consequently degraded, by the discrimination attempted in the bill. It is invidious to select living characters, but it may not be irrelevant to state, that those celebrated physicians Perceval, Currie, and Poultney, would have been disqualified under the act; and yet it may be presumed that neither the late nor present president of the Royal College would venture to dispute with any of them for medical preeminence. The graduates of Edinburgh, Glasgow, &c. will be deprived of valuable privileges conferred upon them by their respective universities; and believing as I do that other encroachments are meditated upon the rights of the profession of every order, if the agitated measure be successful, I recommend it to all classes of the faculty to oppose the first endeavour.

In support of my opinion, it will be sufficient to call to the attention of my readers the bill for reforming medical practice, which was circulated some years since by the College, and which is commonly imputed to the gentleman now at the head of this ancient corporation. Should the present effort be crowned with success, the rising faculty will be led to enter freely into their respective corporations, to qualify themselves to discharge the lucrative offices appointed in the bill. By these means the medical corporations will be gradually extended over the imperial dominions, without undergoing the regulations and improvements which the honor of the profession and responsibility of its members render indispensably necessary. I anxiously wish, for the credit of medical men, and good of society, that the curative art and its various members were placed on the high ground which their laborious avocations and important duties obviously deserve. The termination of war will afford greater leisure to examine our social and domestic establishments. Among these it is to be hoped that the very dangerous state of medical practice will soon obtain its full share of public attention. Let the faculty of every description immediately associate in London on liberal principles, and by uniting their interests with the country practitioners, draw forth an overwhelming opposition to the present bill, partly to defeat the meditated encroachments, partly to substitute measures useful, honourable, and advantageous to the faculty and their employers. C. HARRISON, M.D.

*May* 22, 1814.

P.S.—In the hasty perusal of the bill alluded to, I am aware that several circumstances important to the public and medical men, remain unnoticed by me. Some of the clauses affecting keepers and medical attendants upon lunatic asylums are, in my estimation, peculiarly objectionable; but I have, at present, no leisure to lengthen the inquiry. An early insertion of the "amended bill" would, I conceive, be an acceptable compliment to your readers.

---

*For the Medical and Physical Journal.*

